# Multiple hunting displays in wild broadclub cuttlefish

**DOI:** 10.1002/ecy.70021

**Published:** 2025-02-18

**Authors:** Martin J. How, Cedric van den Berg, Michael Karcz, Charlie Heatubun, Matteo Santon

**Affiliations:** ^1^ School of Biological Sciences University of Bristol Bristol UK; ^2^ Regional Research and Innovation Agency (BRIDA) Manokwari West Papua Indonesia; ^3^ Faculty of Forestry University of Papua Manokwari West Papua Indonesia

**Keywords:** cephalopod, cuttlefish, dynamic crypsis, hunting display, motion camouflage, motion masquerade, predation, sensory exploitation, vision

Predators can make use of a range of camouflage strategies to hunt prey. Ambush predators such as scorpionfish, puff adders, and ghost mantids make use of aggressive crypsis, masquerade, or mimicry to trick prey into approaching close enough to be captured (Glaudas & Alexander, 2017; John et al., 2023, 2024; Skelhorn, 2018). Pursuit predators, such as trumpetfish, falcons, and dragonflies, must deploy other strategies such as dynamic crypsis, motion masquerade, and motion camouflage to conceal their approach from prey, but these are much less well studied (Kane & Zamani, 2014; Mizutani et al., 2003, Matchette et al., 2023; reviewed by Pembury Smith & Ruxton, 2020). One group of shallow‐water species that make use of a prey‐pursuit strategy is the *Sepia* genus of cuttlefish. These species are well known for their remarkable ability to camouflage (Hanlon & Messenger, 2018). A unique level of control of their coloration and texture via muscularly activated chromatophores and extendable papillae provides cuttlefish with the ability to modify their appearance relative to their fine‐scale environment and behavioral context (Allen et al., 2009; Gonzalez‐Bellido et al., 2018; How & Santon, 2022; Messenger, 2001; Osorio et al., 2022; Reiter et al., 2018). How these changes in coloration, texture, and body posture may hinder prey from detecting or recognizing the hunting cuttlefish has not been extensively studied (How et al., 2017; Santon et al., 2025).

Cuttlefish use a strategy of stealth to pursue prey (Messenger, 1968). First, a prey item (usually a small fish or crustacean) is identified and localized using the cuttlefish's monochromatic and polarization‐sensitive visual system (Chung & Marshall, 2016; Groeger, 2004; Marshall & Messenger, 1996; Shinzato et al., 2018; Temple et al., 2021). The cuttlefish then rotates its head and body position to face directly toward the target, employing stereopsis cues to estimate the precise position of the prey (Feord et al., 2020; Wu et al., 2020). It then adopts a hunting display to approach within range to use one of two capture strategies. The most common strategy is the “tentacular strike,” in which the two long tentacles are rapidly extended to attach suckers to the prey (Omura & Ikeda, 2022), which is then pulled back to the buccal opening for consumption. Some species also adopt “jump‐on” or “arm‐grab” behavior, in which all four arm pairs are employed in grabbing the prey item, usually when space is constrained or depending on prey size and type (Adamo et al., 2006; Jiun‐Shian Wu & Chiao, 2023). During the approach phase of hunting, cuttlefish adjust their coloration, texture, and posture. For example, some species raise or wave arms, darken their skin, or express deimatic patterns (Adamo et al., 2006; Kim et al., 2022; Zoratto et al., 2018). Until now, these displays have been described as relatively constrained and species specific, with minor differences more linked to individual personality than context (Zoratto et al., 2018). While some of these display changes are associated with the presence of predators (Adamo et al., 2006), most of the variation in displays during the approach phase of hunting has not yet been investigated, particularly in the wild.

Here, we show that, for the approach phase of prey capture, broadclub cuttlefish *Sepia latimanus*—a large species of cuttlefish that inhabits shallow‐water reefs of the tropical Indo‐pacific region—make use of at least four distinct displays, each of which is remarkably different in coloration, texture, and body posture (Figure 1; Video S1). Across all sequences filmed in the wild (*N* = 234) when presented with live prey crabs (Appendix S1: Section S1), cuttlefish (98 individuals, 40 male and 58 female) first approached from distance. Then, when within approximately 1–2 m of the target, cuttlefish adopted one of four main hunting displays (Figure 2; Video S1).

**FIGURE 1:**
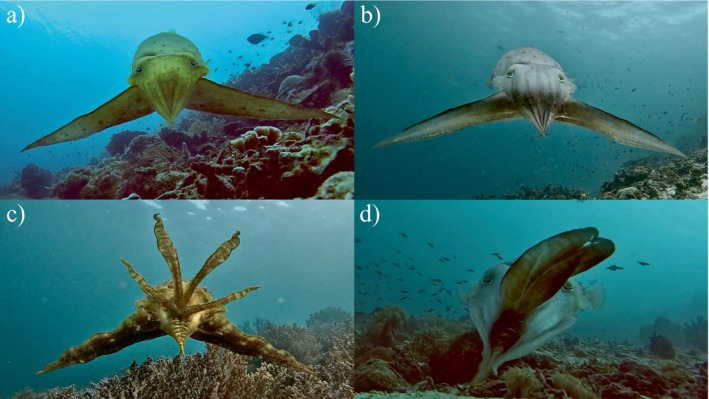
Wild broadclub cuttlefish *Sepia latimanus* hunting with four different displays. Images are from an action camera positioned behind the crab prey. The cuttlefish adopts either the (a) leaf, (b) passing‐stripe, (c) branching coral, or (d) pulse display while approaching the crab. Observations were conducted in the waters off Kri and Mansuar Islands in the Raja Ampat region of Indonesia. Photo credits: Matteo Santon.

**FIGURE 2:**
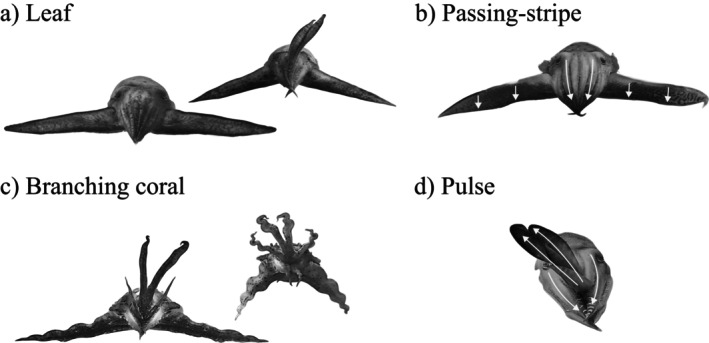
Multiple hunting displays in broadclub cuttlefish. (a–d) Representative examples of the four main hunting displays viewed from prey perspective. When present, insets show alternative postures. Displays can be distinguished by a combination of number of arms raised, body posture, and presence of dynamic dark coloration (passing direction indicated by white arrows). Scheme credits: Matteo Santon.

*Leaf* (Figure 2a): The lateral pair of arms is stretched horizontally with their broad surface presented to the crab. The remaining arms are tucked tightly into a forward‐facing cone, occasionally with the central arm pair raised (e.g., Figure 2a inset). The cuttlefish adopt a static olive green to white coloration with occasional shading along the lower border of the lateral arm pair. The approach tends to be extremely slow and includes dorso‐ventral oscillations in swimming trajectory.

*Passing‐stripe* (Figure 2b): Similar body posture to the leaf display but with an overall gray coloration overlaid with dynamic downward‐moving black stripes (see How et al., 2017; Santon et al., 2025 for further description). The approach trajectory tends to be directed straight toward the prey.

*Branching coral* (Figure 2c): The two central arm pairs are raised, and the lateral arm pair stretched dorso‐laterally, occasionally held smooth or with regular kinks in the arms (e.g., Figure 2c inset). The remaining arm pair is sometimes directed toward the prey, sometimes held downward in a ventral direction. The body adopts one of a range of static color patterns which includes pale or dark coloration with dark mottles. The approach can be direct and fast but also very slow and coupled with lateral oscillation of the raised central arm pair.

*Pulse* (Figure 2d): The two lateral arm pairs are pointed forward in a tight cone, and the two central arm pairs extended diagonally upward and to the side with their broad surface facing prey. The overall body coloration is gray, with pulses of dark color passing slowly from the mantle behind the head to the tips of the raised arm pairs, which can also move up and down synchronously with the passing pulse. The approach tends to be slow and direct.

*Mixed displays*: Not all the 234 displays could be categorized into the four main display types described above. On some occasions, cuttlefish were observed switching between displays during the same hunting approach, for example, starting with a branching coral display and ending with a passing‐stripe display. Also, on some rare occasions, cuttlefish performed hybrid displays, for example, adopting a leaf posture with olive coloration but passing weak dark pulses of color down the arms.

Overall, cuttlefish performed the branching coral, passing stripe and leaf display more frequently, with a probability of occurrence between 22% and 29% (Figure 3a; Appendix S1: Section S2). Pulse and mixed displays occurred less frequently, with occurrence between 11% and 13%. Males and females showed a similar probability of using the different hunting displays (Figure 3b), except from the leaf, which occurred 13% (95% compatibility interval: 4%–22%) more frequently among females. When hunting purple mangrove crabs *Metopograpsus frontalis* (a well armored species with large claws), cuttlefish used the branching coral display 12% (95% compatibility interval: 0.5%–23%) more often than when hunting mottled crabs *Grapsus albolineatus* (a species with softer carapace and smaller claws) (Figure 3c). The other hunting displays occurred with similar frequencies regardless of prey type (Figure 3c). Most cuttlefish (*N* = 62 out of 98) were filmed more than once, with a few up to nine times (Figure 3d). Among the individuals filmed more than once, 49 (79%) performed two or more unique hunting display types (Figure 3e) (How et al., 2025). This suggests that this variability in hunting displays is unlikely explained by individual cuttlefish personality. Furthermore, this great individual variability shows that cuttlefish, in different environmental contexts in the wild, show a much broader behavioral repertoire than when housed in the laboratory. Indeed, captive animals may reduce their behavioral flexibility or develop individual preferences due to oversimplified housing environments and boredom, which is known to affect animal behavior and physiology (Burn, 2017; Crates et al., 2023).

**FIGURE 3:**
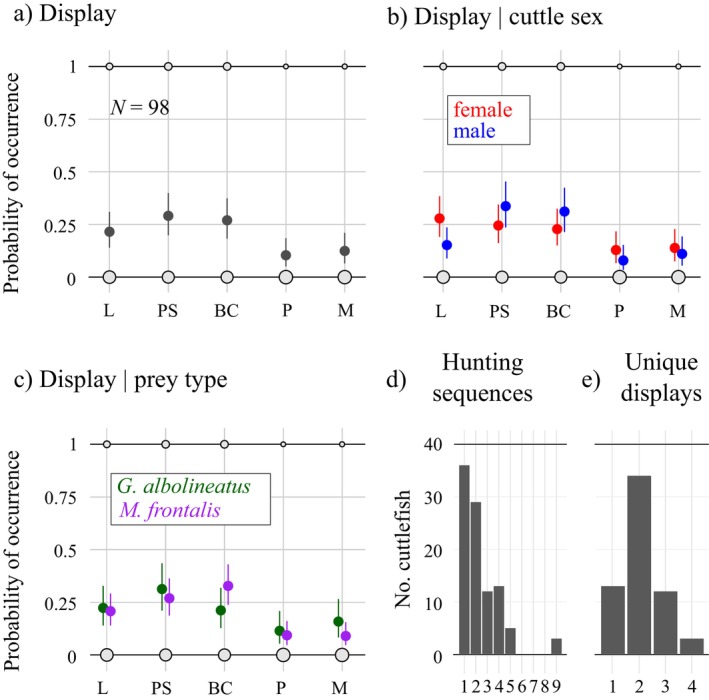
Hunting displays performed by cuttlefish. Model posterior predictions of median probability of occurrence of each display type (solid dots) and its 95% compatibility intervals (error bars) across (a) all sequences filmed, (b) for each cuttlefish sex, and (c) for each prey crab species. Raw data counts are mapped to point area (light gray solid circles) in each panel. BC, branching coral; L, leaf; M, mixed; P, pulse; PS, passing‐stripe. (d) Number of cuttlefish filmed hunting one or more times and (e) for those filmed more than once, number of unique display types shown.

It is compelling to think that each display may hinder the detection or recognition of the approaching cuttlefish by the prey using different mechanisms. Indeed, Santon et al. (2025) recently showed that the passing‐stripe display is a form of motion camouflage, whereby the threatening expanding motion of the approaching predator is overwhelmed by the nonthreatening downward motion generated by the rhythmic stripes. The observed variability between the displays suggests the use of other camouflage strategies than the motion camouflage of the passing‐stripes.

For example, when using the leaf display, cuttlefish adopt a greenish color and perform slow dorso‐ventral swimming oscillation during approach, reminiscent of the movement of a mangrove leaf carried by the current. This display may be a form of motion masquerade, mimicking both the appearance and the movement patterns of innocuous objects in the environment (Pembury Smith & Ruxton, 2020; Skelhorn et al., 2010).

The arm posture adopted by cuttlefish when using the branching coral display is instead ideal to hide among staghorn corals (*Acropora* sp.) or more complex reef backgrounds. This strategy could therefore exploit dynamic crypsis or disruption to hide the approaching cue of the cuttlefish predator (Pembury Smith & Ruxton, 2020).

The mechanism by which the pulse display could improve hunting success is less clear. The body posture adopted by the cuttlefish suggests an attempt to minimize its profile from the perspective of the crab prey, with the exception of the two arm pairs raised above the head and to the side. Viewed by the prey, each chromatic pulse produces a dorso‐laterally directed motion signature along these raised arms, which may simulate the movement of a small nonthreatening fish, thereby masking the expanding loom of the predator with a form of motion masquerade, or simply disrupt prey's attention (Cuthill et al., 2019).

The occasional mixed displays, consisting of partial elements from two different display types, suggest an impressive level of flexibility in the use of these hunting displays. This could be adaptive, with mixed displays offering partial benefits from each component, or could simply represent indecision on the part of the hunting cuttlefish.

Finally, it is important to note that some of these displays (e.g., leaf and branching coral) may also have an additional defensive function, camouflaging the hunting cuttlefish from both the visual perspective of their prey and predators. The extraordinary flexibility of cuttlefish to adopt different coloration, textures, and postures when hunting offers a unique opportunity to study the mechanisms of camouflaging in motion in pursuit predators and presents potential novel solutions to the challenge of remaining undetected while approaching prey. Future research should uncover the fine‐scale contexts in which each of the hunting displays are used to determine which factors influence display choice. It is possible that differences in animal size, habitat use, prey type, or behavior may be driving the choice of a particular hunting display. Alternatively, the cuttlefish may benefit more generally from expressing a diversity of hunting displays to reduce the risk that prey species may learn to avoid the predator or evolve any counter‐adaptation (Dawkins et al., 1997). In this case, the rate of occurrence of display types may fluctuate depending on frequency‐dependent selection driven by prey avoidance behavior (Bond & Kamil, 2002).

## AUTHOR CONTRIBUTIONS

Martin J. How and Matteo Santon conceptualized the project and established underwater methodology. Martin J. How, Cedric van den Berg, Michael Karcz, and Matteo Santon collected the data. Charlie Heatubun is the Indonesian research counterpart and provided logistical support for fieldwork. Matteo Santon analyzed the data. Martin J. How and Matteo Santon wrote the manuscript. All authors reviewed and approved the manuscript.

## FUNDING INFORMATION

This research received the following financial support: Marie Skłodowska‐Curie postdoctoral fellowship 101066328 funded via the Marie Skłodowska‐Curie postdoctoral fellowship 101066328 ‐ Engineering and Physical Sciences Research Council grant EP/X020819/1 (Matteo Santon), Konishi Neuroethology Research Award (Matteo Santon), and Royal Society Fellowship URF\R\201021 and grant RF\ERE\210260 (Martin J. How).

## CONFLICT OF INTEREST STATEMENT

The authors declare no conflicts of interest.

## Supporting information


Appendix S1.

Video S1.

Video S1 Metadata.

## Data Availability

Data (How & Santon, 2025) needed to replicate the analyses and figures presented in this manuscript are available from the University of Bristol data repository: https://doi.org/10.5523/bris.uvmyeefvdq652b9v7pgjrwejh.
