# Supplementary material for: Multiple hunting displays in wild broadclub cuttlefish
Source: Ecology. 2025 Feb 18;106(2):e70021. doi: 10.1002/ecy.70021 (PMC11834760; doi:10.1002/ecy.70021)
Supplement: Supplementary file 1 — Appendix S1. [file ECY-106-e70021-s002.pdf]

Ecology

Appendix S1 for

Multiple hunting displays in wild broadclub cuttlefish

Martin J. How, Cedric van den Berg, Michael Karcz, Charlie Heatubun and Matteo Santon

This file includes:

Section S1: Supplementary methods

Section S2: Supplementary Tables S1-S2

Section S3: Supplementary references

Section 1: Supplementary Methods

Experimental animals

All procedures involving animals followed British and Indonesian laws and directives. Observations were carried out under the research permits (63-64-65-66/SIP/IV/FR/2/2023) issued by the Indonesian National Research and Innovation Agency (BRIN) and the UIN permit 23-003 issued by the University of Bristol. The broadclub cuttlefish *Sepia latimanus* is a large species of cuttlefish that inhabits shallow-water reefs of the tropical Indo-pacific region where it is known to demonstrate a wide repertoire of camouflage and signaling behavior (Corner and Moore 1980). This species was observed hunting after being presented with live prey in its natural environment. Prey species consisted of purple mangrove crabs *Metopograpsus frontalis*, a species with a rather hard carapace and large claws, or mottled crab *Grapsus albolineatus*, a species with softer carapace and smaller claws.

Underwater procedure

265 hunting sequences of wild broadclub cuttlefish were filmed between May 2023 and May 2024 in the shallow waters off Kri and Mansuar Islands in the Raja Ampat region of eastern Indonesia (approx. 0° 33'S 130° 38'E). Researchers diving on self-contained breathing apparatus (SCUBA), on reef depths ranging from 1 to 25m, presented the cuttlefish with live prey consisting of a single crab tethered to a ~50cm string of organic cotton. Crabs had been collected earlier by hand and carried underwater in individual perforated plastic containers. The hunting sequences were filmed from the rough perspective of the hunted crab using an action camera (Hero 11, GoPro, USA) under natural lighting, recording at 240 frames per second at a resolution of 2.7k pixels. Only sequences in which cuttlefish identity could be assigned, based on body size, sex, location and distinctive markings, were retained for analysis (N = 234), resulting in a total of 98 individuals (40 male and 58 female). Hunting displays were categorized using a combination

of number of arms raised or extended, presence and type of dynamic skin pattern, coloration and approach trajectory (details for each display category are outlined in the results).

Statistical analysis

All data analysis was conducted in R v4.3.2 (R Core Team 2023) with the brms package (Bürkner et al. 2016, Bürkner 2017, 2018) which fits Bayesian models using Hamiltonian Monte Carlo via Stan (https://mc-stan.org/). For the linear model, we run 4 chains and obtained coefficient estimates from a total of 8,000 post-warm-up samples. Models were run using within-chain parallelization implemented with the *cmdstanr* package. Visual inspection of trace plots, Monte Carlo standard error, effective number of samples and R-hat values indicated model convergence. Models were further assessed using posterior predictive model checking, which compares model predictions with observed data.

To compare the frequency of occurrence of each display type between cuttlefish sexes and crab species, we modelled the presence or absence of each display type for each filmed sequence using a Bernoulli distribution with logit-link. The model included the categorical predictors *display type* (*leaf, passing stripe, branching coral, pulse, mixed*), *cuttlefish sex* (*female, male*) and *crab species* (*G. albolineatus, M. frontalis*), and the interaction terms between *display type* and each of the two other categorical predictors. We further included the random intercept term sequence_ID (N = 234) and cuttlefish_ID (N = 98) to account for the repeated observations of each sequence and cuttlefish individual. The model was implemented using weakly informative prior distributions [normal with mean = 0 and S.D. = 1.5 for the coefficients of each level of the categorical predictors, exponential(1) for the standard deviations of the multilevel hyperparameters]. For graphical display, we present median response likelihoods and their 95% Compatibility Intervals (CIs) of the posterior distribution of fitted values for the population

average for each level of the categorical predictors of interest (Santon et al. 2023). We further present numerical contrasts (median differences and their 95% CIs) between response likelihoods predicted by the model for each comparison of interest.

Section S2: Supplementary Tables S1-S2

Table S1. Pairwise contrasts between the probability of occurrence of each display type across all sequences filmed expressed as median difference and its 95% CIs.

**Comparison**	**Difference**	**95 % CI**	
		**Low**	**High**
Leaf display – Passing stripe display	-0.08	-0.15	0.01
Leaf display – Branching coral display	-0.05	-0.13	0.03
Leaf display – Pulse display	0.11	0.04	0.18
Leaf display – Mixed display	0.09	0.02	0.16
Passing stripe display – Branching coral display	0.02	-0.06	0.10
Passing stripe display – Pulse display	0.18	0.11	0.26
Passing stripe display – Mixed display	0.16	0.09	0.24
Branching coral display – Pulse display	0.16	0.10	0.23
Branching coral display – Mixed display	0.14	0.07	0.21
Pulse display – Mixed display	-0.02	-0.08	0.04

Table S2. Parameter estimates for model investigating the likelihood of display type occurrence between cuttlefish sexes when presented with two different prey items.

Parameters	Mean	M. Error	95 % CI	
			Low	High
**Regression coefficients**				
Leaf display	-0.91	0.28	-1.47	-0.38
Passing stripe display	-1.03	0.28	-1.58	-0.49
Branching coral display	-1.55	0.31	-2.19	-0.97
Pulse display	-1.82	0.38	-2.61	-1.11
Mixed display	-1.55	0.34	-2.27	-0.90
Male cuttlefish	-0.78	0.30	-1.37	-0.19
Purple crab	-0.09	0.30	-0.68	0.50
Passing stripe display, Male cuttlefish	1.23	0.40	0.44	2.03
Branching coral display, Male cuttlefish	1.21	0.41	0.40	2.02
Pulse display, Male cuttlefish	0.23	0.50	-0.75	1.20
Mixed display, Male cuttlefish	0.52	0.48	-0.44	1.47
Passing stripe display, Purple crab	-0.13	0.41	-0.95	0.69
Branching coral display, Purple crab	0.70	0.43	-0.15	1.54
Pulse display, Purple crab	-0.13	0.51	-1.13	0.88
Mixed display, Purple crab	-0.56	0.48	-1.51	0.41
**Multilevel Hyperparameters for sequence ID, N = 234**				
Sd (Intercept)	0.06	0.05	0.00	0.17
**Multilevel Hyperparameters for cuttlefish ID, N = 98**				
Sd (Intercept)	0.06	0.04	0.00	0.17

Section S3: Supplementary references

Bürkner, P.-C. 2017. brms: An R package for Bayesian multilevel models using Stan. Journal of statistical software 80:1–28.

Bürkner, P.-C. 2018. Advanced Bayesian multilevel modeling with the R package brms. R J. 10, 395–411. doi: 10.32614. RJ-2018-017.

Bürkner, P.-C., J. Gabry, and S. Weber. 2016. brms: Bayesian regression models using Stan. R package version 1.

Corner, B. D., and H. T. Moore. 1980. Field observation on the reproductive behavior of *Sepia latimanus*. Micronesica 16:235–260.

R Core Team. 2023. R: A language and environment for statistical computing. R Foundation for Statistical Computing, Vienna, Austria. URL: https://www.R-project.org/.

Santon, M., F. Korner-Nievergelt, N. K. Michiels, and N. Anthes. 2023. A versatile workflow for linear modelling in R. Frontiers in Ecology and Evolution 11.
