# Supplementary material for: Multiple hunting displays in wild broadclub cuttlefish
Source: Ecology. 2025 Feb 18;106(2):e70021. doi: 10.1002/ecy.70021 (PMC11834760; doi:10.1002/ecy.70021)
Supplement: Supplementary file 3 — Video S1 Metadata. [file ECY-106-e70021-s003.pdf]

Video S1 metadata for

Multiple hunting displays in wild broadclub cuttlefish

Martin J. How, Cedric van den Berg, Michael Karcz, Charlie Heatubun and Matteo Santon

Video S1. Wild broadclub cuttlefish *Sepia latimanus* hunting with four different displays. The cuttlefish adopts either the *leaf*, *passing-stripe*, *pulse*, or *branching coral* display (clockwise from top left) while approaching prey crab. Footage was collected in the waters off Kri and Mansuar Islands in the Raja Ampat region of Indonesia. Videos were synchronized to the final strike at prey. Video credits: Matteo Santon.
